# Human Papillomavirus Infections and the Role Played by Cervical and Cervico-Vaginal Microbiota—Evidence from Next-Generation Sequencing Studies

**DOI:** 10.3390/cancers16020399

**Published:** 2024-01-17

**Authors:** Maria Głowienka-Stodolak, Katarzyna Bagińska-Drabiuk, Sebastian Szubert, Ewa E. Hennig, Agnieszka Horala, Michalina Dąbrowska, Martyna Micek, Michał Ciebiera, Natalia Zeber-Lubecka

**Affiliations:** 1 Department of Genetics, Maria Sklodowska-Curie National Research Institute of Oncology, 02-781Warsaw, Poland; maria.glowienka@nio.gov.pl (M.G.-S.); katarzyna.baginska@nio.gov.pl (K.B.-D.); ehennig@cmkp.edu.pl (E.E.H.); michalina.dabrowska@nio.gov.pl (M.D.); 2 Division of Gynaecological Oncology, Department of Gynaecology, Poznan University of Medical Sciences, 61-701 Poznan, Poland; sszubert@ump.edu.pl (S.S.); ahorala@ump.edu.pl (A.H.); 3 Department of Gastroenterology, Hepatology and Clinical Oncology, Centre of Postgraduate Medical Education, 02-781 Warsaw, Poland; 4 Second Department of Obstetrics and Gynecology, Centre of Postgraduate Medical Education, 00-189 Warsaw, Poland; micekm@bielanski.med.pl (M.M.); mciebiera@cmkp.edu.pl (M.C.); 5 Warsaw Institute of Women’s Health, 00-189 Warsaw, Poland

**Keywords:** human papillomavirus (HPV) infections, cervical microbiota, cervico-vaginal microbiota, next-generation sequencing (NGS) technology, *16S rRNA* gene, cervical intraepithelial neoplasia (CIN), squamous intraepithelial lesion (SIL)

## Abstract

**Simple Summary:**

This review explores the impact of cervical microbiome changes on human papillomavirus (HPV) using next-generation sequencing (NGS). HPV poses global health concerns, from benign lesions to cervical cancer. The cervical microbiome, a unique microorganism collection in the cervix, is crucial for cervical health. Recent research suggests that disruptions in the cervical microbiome, marked by reduced *Lactobacillus* and bacterial overgrowth, may heighten HPV persistence and cervical abnormalities. NGS technology has transformed cervical microbiome studies, revealing insights into microbial diversity and dynamics. Bacterial *16S rRNA* gene sequencing proves valuable in understanding the cervical microbiome’s role in HPV infections. NGS-based studies provide personalized insights into individuals’ cervical microbiomes, holding promise for novel diagnostic tools, therapies, and preventive interventions for cervical conditions, including cancer. The research aims to enhance global women’s health through a comprehensive understanding of the cervical-microbiome–HPV relationship.

**Abstract:**

This comprehensive review encompasses studies examining changes in the cervical and cervico-vaginal microbiota (CM and CVM) in relation to human papillomavirus (HPV) using next-generation sequencing (NGS) technology. HPV infection remains a prominent global health concern, with a spectrum of manifestations, from benign lesions to life-threatening cervical cancers. The CM and CVM, a unique collection of microorganisms inhabiting the cervix/vagina, has emerged as a critical player in cervical health. Recent research has indicated that disruptions in the CM and CVM, characterized by a decrease in *Lactobacillus* and the overgrowth of other bacteria, might increase the risk of HPV persistence and the progression of cervical abnormalities. This alteration in the CM or CVM has been linked to a higher likelihood of HPV infection and cervical dysplasia. NGS technology has revolutionized the study of the cervical microbiome, providing insights into microbial diversity, dynamics, and taxonomic classifications. Bacterial *16S rRNA* gene sequencing, has proven invaluable in characterizing the cervical microbiome, shedding light on its role in HPV infections and paving the way for more tailored strategies to combat cervical diseases. NGS-based studies offer personalized insights into an individual’s cervical microbiome. This knowledge holds promise for the development of novel diagnostic tools, targeted therapies, and preventive interventions for cervix-related conditions, including cervical cancer.

## 1. Introduction

Human papillomaviruses (HPVs) are a group of viruses that may infect the skin and mucous membranes of various body parts, including the cervix [1]. It is one of the most common sexually transmitted infections worldwide. Among nearly 200 types of HPV, high- and low-risk (hr and lr) types were distinguished for the development of cancerous lesions [2]. Currently, 14 hrHPV oncogenic virus types, including types 16, 18, 31, 33, 35, 39, 45, 51, 52, 56, 58, 59, 66, and 68, are causally associated with cancer development [1,3]. A persistent infection with oncogenic types may result in cancer of the cervix, anus, vagina, vulva, penis, and the throat. HPV-16, HPV-18, HPV-31, HPV-33, and HPV-58 are most commonly identified in cervical cancer cells, with HPV-16 and HPV-18 being found in over 70% of cervical cancer [4]. In turn, lrHPV types, including HPV-6, HPV-11, HPV-40, HPV-42, HPV-43, HPV-44, HPV-54, HPV-61, HPV-72, and HPV-82, are responsible for the development of benign papillomatous lesions of the mucous membranes and skin. In practice, these include genital warts and recurrent papillomatosis of the larynx [5,6]. HPV is primarily transmitted through sexual contact, and factors such as multiple sexual partners, early sexual activity, and a weakened immune system may increase the risk of infection [7]. Most HPV infections are asymptomatic and transient. However, in some cases, when spontaneous clearance of the virus is not achieved, potentially serious or even life-threatening diseases develop, including cervical cancer. The overwhelming number of infections with various types of HPV resolve spontaneously due to the body’s natural immune response. A persistent HPV infection (i.e., infections lasting >24 months) may lead to oncogenesis in subsequent years [7].

## 2. HPV Infection and Cervical Cancer

Cervical cancer (CC) is a significant global health issue, and the burden of the disease is particularly high in low- and middle-income countries [8]. CC is the fourth most common cancer in women worldwide [9]. About 604,000 women develop it annually, of whom about 60% die. The peak incidence is between the ages of 50–60 [10]. HPV viruses show tropism to the epithelial cells of the mucous membranes and skin. Tropism varies depending on the type of virus. Virions penetrate the basal layer of the epithelium, while the assembly and release of progeny virions take place in the upper layers of the epithelium [11]. A productive viral replication cycle requires the viral oncoproteins E6 and E7, which create a favorable environment for viral DNA replication in the middle layers of the epithelium, where DNA replication would not normally be possible [12]. The HPV genome undergoes integration at the reading frame breakpoint for the E2 protein, which controls the expression of E6 and E7. The absence of the E2 protein leads to an increased synthesis of both oncogenic proteins, and their excessive activity, to the neoplastic transformation of the infected cell. In turn, the overexpression and activity of viral oncoproteins E6 and E7 lead to the deregulation of the cell cycle, increased cell division, inhibition of apoptosis, and the accumulation of genetic damage due to inefficient DNA repair, resulting in the development of tumorigenesis [13]. Regular cervical cancer screening, such as Pap smears and HPV testing, is essential for early detection and timely intervention to prevent cervical cancer and its complications [14,15,16]. HPV vaccination is a highly effective preventive measure against HPV infection and its associated diseases, including cervical cancer. Vaccination can protect against the most common hrHPV types and is recommended for both boys and girls before they become sexually active [17].

Lesions referred to as low- or high-grade cervical intraepithelial neoplasia (CIN) are far more common in the cervix [18]. CIN is a precancerous condition that also results from a persistent infection with HPV within cervical cells [19]. CIN is classified into three grades, CIN-1, CIN-2, and CIN-3, based on the degree of abnormality in the cervical cells [18]. CIN-1 represents mild dysplasia and is often associated with low-grade squamous intraepithelial lesions (LSIL) on Pap smears. In young women, CIN-1 lesions commonly regress to normal without treatment due to the body’s intact immune response and the cervical rapid cell turnover [18]. Approximately 60% of CIN-1 cases regress to normal within one year. In turn, CIN-2 and CIN-3 represent moderate-to-severe dysplasia, and they carry a higher risk of progressing to invasive cervical cancer compared to CIN-1. However, the average time for progression to invasive cancer is still several years [20].

## 3. Relationship between Vaginal and Cervico-Vaginal Microbiota and HPV Infection

Research findings indicate that the microbiota across distinct segments of the female genital tract may share similarities while displaying variations [21,22,23]. These differences are observed as one moves from the vagina to the cervix, endometrium, fallopian tubes, and peritoneal fluid. The prevailing trend in most studies has been to label samples as cervico-vaginal rather than explicitly addressing and distinguishing between cervical and vaginal samples.

The vaginal microbiota (VM), cervical microbiota (CM), and cervico-vaginal microbiota (CVM) describe a collection of microorganisms, including bacteria, viruses, and fungi, that reside in vagina and on the cervix. Various factors may influence the composition of the VM, CM, and CVM, including hormonal changes, sexual activity, hygiene practices, and contraceptive and antibiotic use [24,25,26]. The composition of the CVM may vary among individuals but is generally dominated by *Lactobacillus* species, which are considered beneficial bacteria [27]. The most common *Lactobacillus* species found in the vagina and cervix include *Lactobacillus crispatus*, *Lactobacillus gasseri*, *Lactobacillus jensenii*, *Lactobacillus acidophilus*, and *Lactobacillus iners* [27,28,29]. Lactic acid, a metabolic byproduct of fermenting sugars produced by lactobacilli, helps maintain a balanced pH, prevents the overgrowth of harmful microorganisms, and supports the local immune system [30]. The VM and CVM play a crucial role in maintaining the vaginal and cervical condition.

Microbial communities in the vagina and cervico-vaginal environment have been classified into five major community status types (CSTs). However, the current CST classification provides only a partial understanding of the relationship between the microbiota and cervico-vaginal conditions in women. The limitations of the bacterial identification technologies used contribute to that understanding [31]. Previously, Ravel et al. identified four vaginal different types of community status (CST): I, II, III, and V [32]. These correspond to the microbiota, showing a predominance of specific *Lactobacillus* species. CST-I was dominated by *L. crispatus*, CST-II by *L. gasseri*, CST-III by *L. iners*, and CST-V by *L. jensenii*. In contrast, CST-IV presented a diverse microbial composition. France et al. introduced the VALENCIA (vaginal community state type nearest centroid) classifier tool for consistent assignment of CSTs within the VM of reproductive-age women [33]. VALENCIA’s applicability was validated on diverse datasets, including reproductive-age women from eastern and southern Africa, adolescent girls, and a diverse group of postmenopausal women. Despite variations in sequencing and bioinformatics, VALENCIA performed well, demonstrating its broad applicability for VM classification. Firstly, of the seven identified CSTs, four were rich in *Lactobacillus* species. These CSTs were further categorized into thirteen sub-CSTs. Following the naming convention from previous studies, the authors designated them as CST I (*L. crispatus*-dominated), CST II (*L. gasseri*-dominated), CST III (*L. iners*-dominated), and CST V (*L. jensenii*-dominated). CSTs I and III were more prevalent in the dataset and were subdivided into A and B versions, reflecting variations in the relative abundance of the focal species. Additionally, three CSTs with lower lactobacilli abundance were identified as CST IV-A (high *Candidatus Lachnocurva vaginae* and moderate *G. vaginalis*), CST IV-B (high *G. vaginalis* and low *Candidatus L. vaginae*), and CST IV-C (low *Lactobacillus* spp., *G. vaginalis*, *A. vaginae*, and *Candidatus L. vaginae*). CST IV-C was further divided into five sub-CSTs: CST IV-C0 (even community with a moderate amount of *Prevotella*), CST IV-C1 (*Streptococcus*-dominated), CST IV-C2 (*Enterococcus*-dominated), CST IV-C3 (*Bifidobacterium*-dominated), and CST IV-C4 (*Staphylococcus*-dominated). Depending on the race, self-identifying black or African American women were less likely to have CST I compared to white or Asian women. Black women showed a higher likelihood of having CST IV-A than white women and CST IV-B compared to white or Asian women. Asian women in the study did not exhibit CST IV-A, and CST IV-B was more common among Hispanic women than white women. Asian women were more likely to have CST III than black or white women, although this association was weaker. No significant associations with race were found for CSTs II, V, or IV-C, potentially due to sample size limitations as these three CSTs are less prevalent [33].

Emerging research suggests that the composition of the CVM may influence an individual’s susceptibility to HPV infections and the subsequent development of cervical lesions or cancer [26]. Disruptions in the normal cervical microbiota, such as a decrease in *Lactobacillus* species or an overgrowth of other bacteria, were associated with an increased risk of HPV persistence and the progression of cervical abnormalities [34]. Specifically, a lower abundance of *Lactobacillus* species and an increased presence of certain types of bacteria, such as *Gardnerella vaginalis*, were associated with an altered CVM and a higher risk of HPV infection and cervical dysplasia [35,36]. Additionally, it was suggested that the CVM might affect the local immune response to HPV infections [37]. Certain bacteria in the microbiome can stimulate immune cells and modulate inflammation, potentially influencing the clearance or persistence of HPV [38,39]. While HPV vaccination has reduced the cervical cancer burden, non-vaccine-preventable HPV types still pose a risk [40]. Notably, not all hrHPV-infected women develop cervical cancer, prompting researchers to explore potential protective factors. One hypothesis suggests that beneficial bacteria, like *L. acidophilus* in the CVM, could contribute to safeguarding against CC development in hrHPV-infected women [41]. Through detailed microbiome profiling in a Dutch CC screening program found that women with typical cervical smears and a higher *L. acidophilus* abundance were associated with a lower risk of HSIL, highlighting the role of this bacteria in cervico-vaginal microbial dynamics and continuity [41].

Moreover, in another study, Molina et al., in analyzing a longitudinal cohort of 141 women diagnosed with hrHPV infection, found that long-term changes in the CVM composition positively correlate with microbial diversity at two timepoints six-months apart [42]. Women with an initial high abundance of *L. iners* tend to have a more stable microbiome composition in subsequent visits compared to those with *Lactobacillus*-depleted communities at baseline. Additionally, specific species such as *L. acidophilus* and *Megasphaera genomosp* type 1 are associated with changes in CSTs between visits. Notably, *Gardnerella vaginalis* was linked to the stability of *Lactobacillus*-depleted communities, while *L. iners* was associated with the instability of *Megasphaera genomosp* type 1-dominated communities. These findings suggest dynamic CVM patterns during hrHPV infection, offering potential insights for the development of microbiome-based therapies to counter infection progression toward disease [42].

**Figure 1:**
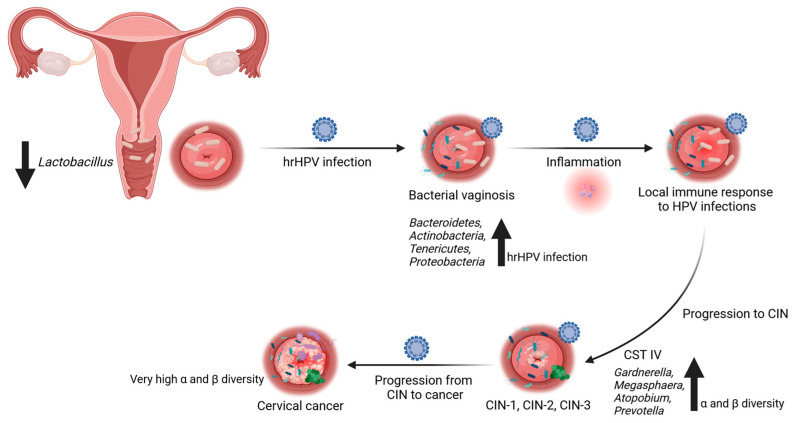
Relationship between cervical and cervico-vaginal microbiota and high-risk HPV infections. The composition of cervical microbiota can impact vulnerability to high-risk (hr) HPV infections and the subsequent development of cervical lesions or cancer. Disturbances in the normal cervical microbiota, a reduction in *Lactobacillus* species, or an overgrowth of other bacteria are linked to an elevated risk of persistent HPV infection and the advancement of cervical abnormalities. Bacterial vaginosis (BV) is a shift from the dominance of *Lactobacillus* to a more diverse microbiome characterized by increased levels of anaerobic bacteria like *Gardnerella vaginalis*, *Peptostreptococcus anaerobius*, and *Porphyromonas uenoni*. BV is associated with an increased susceptibility to HPV-related cervical intraepithelial neoplasia (CIN) and cervical cancer. Decreased abundance of *Lactobacillus* species and increased presence of *Gardnerella vaginalis* correlate with an altered cervical and cervico-vaginal microbiome (CM/CVM) and higher risk of HPV infection and cervical dysplasia. Certain bacteria within the CM and CVM can activate immune cells and regulate inflammation, potentially affecting the clearance or persistence of HPV. As the lesions progress, an upward trend in species diversity is noted. Progression from CIN to cancer requires persistent HPV infection. Created with BioRender.com (accessed on 10 December 2023).

The presence and quantity of lactobacilli in the vaginal microbiome vary with age and are influenced by estrogen levels. Lactobacilli play a crucial role in converting glycogen in the mature vaginal epithelium into organic acids, primarily lactate. This acidification of the vaginal environment creates a protective barrier against viral and bacterial pathogens [43,44]. However, during menopause, when estrogen levels decline, there is a reduction in *Lactobacillus* populations and an increase in anaerobic bacteria in the vaginal flora [39]. Nevertheless, several other factors such as ethnicity, sexual activity, hygiene practices, lactation, and dietary habits may also impact the composition of the vaginal microbiota [38,45]. Changes in the vaginal microbiota may lead to immune regulation and inflammation, which are associated with various gynecological conditions, including bacterial vaginosis [21,46]. Bacterial vaginosis represents a shift from the predominance of *Lactobacillus* to a more diverse microbiome with higher levels of anaerobic bacteria like *Gardnerella vaginalis*, *Peptostreptococcus anaerobius*, and *Porphyromonas uenonis*. Importantly, bacterial vaginosis was linked to an increased risk of HPV-related CIN and cervical cancer [34,47,48] (Figure 1). A well-balanced microbiome was linked to a reduced risk of infections, including bacterial vaginosis and urinary tract infections [49].

Radiation therapy and chemoradiation therapy, whether used for curative or palliative purposes in gynecologic cancers, may impact the composition of the CM and CVM [50]. In a small-scale study, it was observed that radiation therapy led to a significant decrease in the abundance of cervical bacteria, although there were no discernible changes in the bacterial alpha- or beta-diversity [44]. Another investigation revealed 13 phylogroups at the genus level that differentiated the cervical microbiota before and after radiation therapy. Furthermore, most of the post-radiation therapy microbiota communities were distinct from those found in a healthy, normal microbiome. Another study indicated a tendency toward lower microbial richness in samples collected from healthy individuals compared to those from patients with gynecological cancer [51]. In a self-reported study, we showed an increased diversity of the CM associated with cervical cancer [50]. In healthy premenopausal women, *Lactobacillus* dominated in the CM, accounting for over 90% of the microbial community. However, in both pre- and postmenopausal cancer patients before treatment, the CM exhibited a heterogeneous composition, with a lower proportion of *Lactobacillus*, especially in younger patients. At the genus level, we identified taxa that differentiated healthy controls from cancer patients in the pre- and postmenopausal groups, respectively. Furthermore, 31 and 2 genera distinguished pre-radiation from post-radiation samples and pre-radiation from follow-up samples, respectively. Interestingly, microbiome diversity was significantly higher in patients before treatment compared to healthy controls. Such findings highlight significant changes in the CM of cervical cancer patients when compared to healthy controls, with more pronounced alterations occurring after chemoradiation therapy [50]. However, the exact mechanisms underlying the relationship between the CM and HPV infections are still being investigated, and further research is needed to understand the complexities of this interaction fully.

## 4. Microbial Influence on Cervical Cancer Development: Immune Responses and Therapeutic Prospects

The comprehensive analysis of the gastrointestinal (GI) microbiota has significantly advanced comprehension of how the human microbiome influences overall host health. Functions supported by the GI microbiome encompass immune system development, digestion, fat metabolism, epithelial homeostasis, and enteric nerve regulation [52]. Among healthy women, both the gut and vaginal microbiota are shielded from the host by a multi-level barrier system, including a mucus layer, the secretion of soluble immune mediators, and an intact epithelium with tight junctions [53]. Failure of this multifaceted barrier system can lead to the translocation of pathogenic bacteria across the gut and vaginal epithelia, inducing low-grade chronic inflammation and subsequent diseases, including cancer [54]. Conversely, cancers in the GI and reproductive tracts can cause inflammation, resulting in dysbiosis and establishing a positive feedback loop that may contribute to disease promotion [53].

Wang et al. first revealed significant changes in the diversity and composition of the gut microbiota in CC patients [55]. Seven genera, including *Escherichia–Shigella*, *Roseburia*, *Pseudomonas*, *Lachnoclostridium*, *Lachnospiraceae_UCG-004*, *Dorea*, and *Succinivibrio*, exhibited significant differences in relative abundance between CC and controls. Characteristic microbiome features were identified, suggesting a *Proteobacteria* phylum in CC patients as potential biomarkers [55].

In turn, Sims et al. showed a significantly higher alpha diversity in CC patients compared to controls, with this association being more prominent in older women (>50 years) [56]. Age- and race-adjusted LEfSe analysis revealed multiple taxa differences between the two groups, with *Prevotella*, *Porphyromonas*, and Dialister being significantly enriched in CC patients, while *Bacteroides*, *Alistipes*, and members of the *Lachnospiracea* family were significantly enriched in healthy subjects. Importantly, *Prevotella*-rich environments stimulate dendritic cells (DC) through Toll-like receptor 2 (TLR2), releasing interleukin-1b (IL-1b), IL-6, and IL-23. This facilitates IL-17 production by T helper 17 (Th17) cells, activating neutrophils [57]. *Prevotella*’s role in altering host immunity and modulating immunologic pathways may be linked to CC risk and treatment outcomes [56]. Moreover, in a subsequent study, Sims et al. [58] linked gut microbiota diversity and a positive response to chemoradiation in CC patients. The composition variation among patients was associated with both short-term and long-term survival. Short-term survivors exhibited enrichment in *Porphyromonas*, *Porphyromonadaceae*, and *Dialister*, while long-term survivors showed enrichment in *Escherichia Shigella*, *Enterobacteriaceae*, and *Enterobacteriales*. Therefore, modulating the gut microbiota prior to chemoradiation could be a potential avenue to enhance treatment effectiveness and overall outcomes in cervical cancer patients [58].

**Figure 2:**
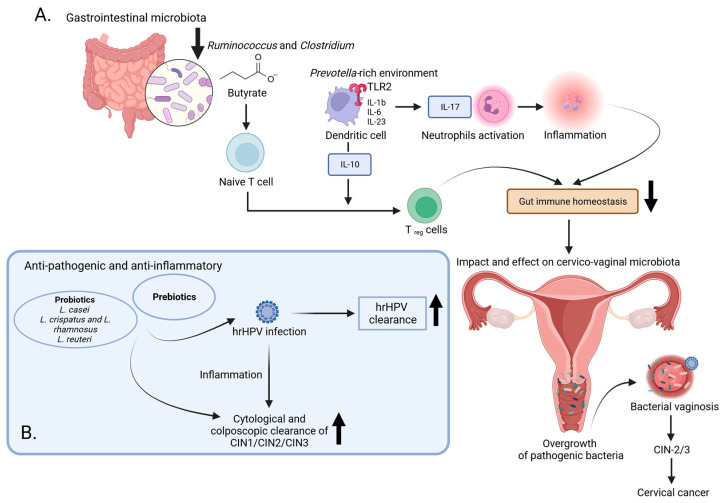
The impact of microbiota on the progression of cervical cancer: examining immune reactions and potential therapeutic avenues. (**A**) In healthy women, the gut and vaginal microbiota are protected by a multi-layered barrier system comprising a mucus layer, immune mediators, and an intact epithelium. Failure of this barrier can lead to the translocation of pathogenic bacteria, causing chronic inflammation and cancer. *Prevotella*-rich environments stimulate dendritic cells via TLR 2, releasing cytokines and promoting immune responses that may be linked to cervical cancer (CC) risk. CC patients show a decrease in butyrate-producing bacteria, essential for controlling inflammation and maintaining intestinal health. Butyrate microbial metabolites also stimulate cells to produce anti-inflammatory compounds, contributing to intestinal homeostasis. The reduction in *Ruminococcus* and *Clostridium* may impact overall intestinal health, thereby influencing vaginal health. (**B**) The use of prebiotics and probiotics has shown promise in preventing HPV-induced cervical malignancy. Results indicated increased rates of HPV clearance, cytological and colposcopic clearance of abnormalities, and improved histological outcomes following treatment. These supplements, known for their positive effects on digestive system function and immune processes, contribute to overall health and may play a role in managing HPV-related cervical lesions. Created with BioRender.com (accessed on 13 January 2024).

In Kang et al.’s study [59], the *Prevotella* genus was significantly more abundant in the CC group and *Clostridium* in the HC group. Additionally, a developed machine-learning-based classifier model differentiated CC from controls in terms of seven bacterial genera, i.e., *Prevotella*, *Peptostreptococcus*, *Finegolida*, *Ruminococcus*, *Clostridium*, *Pseudomonas*, and *Turibacter*. The model exhibited excellent diagnostic performance, providing an effective prediction capability for early invasive CC (ICC). A decrease in butyrate-producing bacteria, including *Ruminococcus* and *Clostridium*, was observed in the CC patient group. Butyrate, a vital nutrient in the intestinal tract, is essential for controlling inflammation, preventing leaky gut, and regulating intestinal autophagy and energy metabolism in the human colon [60]. The reduction in these bacteria may impact overall intestinal health, thereby influencing vaginal health. Chang et al. identified *Ruminococcus 2* as a gut flora family closely linked to CC, suggesting its potential as a biomarker for predicting cervical cancer development [61]. *Firmicutes*, particularly *Ruminococcus*, plays a crucial role in polysaccharide degradation and contributes to human metabolism by converting cellulose into host nutrients [62]. Additionally, *Ruminococcus* is associated with the intestinal barrier, cellular immunity, inflammation, and metabolism [63]. In summary, the connection between *Prevotella*, *Ruminococcus*, and *Clostridium* suggests a potential association with an increased risk of early ICC (Figure 2A) [59].

The factors influencing the outcome of HPV infection and the mechanisms by which the host immune system safeguards against HPV remain elusive [64]. TLRs, a class of pattern recognition receptors located in the cytoplasm and on cell membranes, possess the ability to specifically identify pathogen-associated molecular patterns [65]. As pivotal components of both innate and adaptive immunity, TLRs not only play critical roles in defending against infectious diseases but are also implicated in the initiation and progression of various malignant tumors [66,67]. Polymorphisms within TLR genes have been linked to CC, though certain inconsistencies exist in the reported results [68,69,70]. However the meta-analysis results indicated that carriers of the +1196T (rs4986791 *TLR4*), +7764T (rs1927911 *TLR4*), −1486C (rs187084 *TLR9*), and +2848A (rs352140 *TLR9*) alleles, as well as the −2604G/G (rs10759931 *TLR4*) and −1237C/C (rs5743836 *TLR9*) genotypes, were associated with an elevated risk of CC [71]. Bioinformatics analysis unveiled that the −1237T > C (rs5743836) and −1486T > C (rs187084) polymorphisms could impact transcription factor binding sites (RELA, NFKB1, and THAP1) in the TLR9 gene. Additionally, the +2848G > A (rs352140) polymorphism appeared to alter the structure and stability of the TLR4 protein [71]. These findings suggest that TLR4 and TLR9 gene polymorphisms may influence intracellular signaling pathways, potentially altering immune response patterns and contributing to an increased susceptibility to cervical cancer.

Werner et al. proposed a hypothesis suggesting that the progression of CC might be linked to changes in the expression of innate immune receptors, specifically integrins and TLRs, and that these changes could be induced by infectious agents [72]. Their investigation involved the analysis of protein expression in cervical biopsy tissues and various cervical-cancer-derived cell lines (HeLa, CaSki, SiHa, C-33 A, and ME180). Immunohistochemistry analysis revealed an upregulation of integrin αv, β3, β4, and β6 expression in the epithelium during the development of cervical cancer. Notably, there was a noticeable increase in integrin β6 expression in cell lines containing HPV genetic material compared to the HPV-negative C-33 A cell line. To investigate the potential effects of bacterial infections on TLRs and integrins, HeLa cells were infected with two pathogens, *Escherichia coli* and *Pseudomonas aeruginosa*, while using *Lactobacillus reuteri* as a control. The results indicated that infection with *E. coli* or *P. aeruginosa*, but not with *L. reuteri*, significantly altered the expression of TLRs and integrins, with a notable impact on TLR4 and integrin β6. Considering the pivotal roles of both integrin β6 and TLR4 in tumorigenesis, these findings suggest that bacterial infections may serve as triggers for cancer development in the HPV-infected cervical epithelium.

Another study by Wang et al. focused on elucidating the expression, distribution, and functional activity of TLR4 in normal cervical tissues, CIN, ICC, and various CC cells infected with HPV [73]. The findings revealed a correlation between TLR4 expression and histopathological grade, with a higher expression in ICC compared to CIN and a lower expression in normal cervical tissues and malignant cervical stroma. Moreover, TLR4 expression was elevated in SiHa cells (HPV16+) compared to HeLa cells (HPV18+), while no expression was observed in C33A cells (HPV−). Upon treatment with the TLR4 agonist lipopolysaccharide (LPS), SiHa cells exhibited an increased TLR4 expression and developed resistance to apoptosis, a phenomenon not observed in HeLa or C33A cells. Interestingly, LPS treatment did not alter the cell cycle distribution in SiHa cells. The mechanism behind apoptosis resistance seemed to be linked to HPV-16 infection and was not correlated with changes in cell cycle distribution. Targeting TLR4, especially in combination with traditional drug treatments, could represent a novel strategy for more effectively eliminating cancer cells. This approach holds promise for enhancing the efficacy of cancer therapies by addressing specific molecular pathways associated with TLR4 and HPV infection [73].

The conventional treatment for CIN involves surgical methods, such as ablative or excisional procedures. However, these approaches primarily address the visible lesion without directly targeting the underlying cause associated with HPV infection. Developing a successful approach to address persistent HPV infection or inflammation could significantly impact global health and have widespread economic implications.

The elimination of genital verrucous lesions with imiquimod is likely facilitated by the stimulation of both innate and cellular immunity [74,75]. This involves the initiation of antiviral activity through the induction of cytokines, including interferon-α (IFN-α), tumor necrosis factor-α (TNF-α), and ILs [76]. Imiquimod is known to activate immune cells by interacting with TLR7 on the cell surface, a receptor commonly involved in recognizing pathogens. Activation of cells through imiquimod and TLR7 prompts the secretion of cytokines such as IFN-α, IL-6, and TNF-α, contributing to the antiviral immune response [76]. Topical imiquimod demonstrates both efficacy and tolerability in treating persistent HPV infection, with or without CIN or vaginal intraepithelial neoplasia (VAIN) [77]. Moreover, a weekly topical application of 5% imiquimod cream has demonstrated effectiveness in promoting the regression of HSIL in the cervix, as evidenced by histologic response rates [78]. While imiquimod is considered as a potential treatment for CIN and VAIN, limited attention has been given to investigating the adverse events associated with its vaginal use [78,79,80]. Despite the occurrence of common local and systemic complications, discontinuation of treatment is not a frequent outcome [79]. Consequently, imiquimod holds promise as a potential alternative to surgical interventions for managing CIN [81]. A more comprehensive assessment of imiquimod as a therapeutic option for CIN and VAIN is needed, considering both its potential benefits and the associated risks of adverse events.

At present, the management of CC relies on a collaborative approach involving a multidisciplinary team. In the initial phases of the disease, various treatment modalities are available, encompassing surgery, radiation, neoadjuvant chemotherapy, and procedures for fertility preservation [82]. Concurrent chemoradiation with the use of cisplatin, either alone or in conjunction with other drugs, stands as the conventional therapeutic approach for individuals diagnosed with locally advanced CC [82]. Various therapeutic options exist for the treatment of metastatic patients dealing with lung metastasis, bone metastasis, single brain metastasis, or multiple brain metastases. The growing focus on human health has led to a rapid rise in the therapeutic and commercial interest in producing supplements, including prebiotics and probiotics, due to their association with the microbiota [83,84]. Probiotics play a role in multiple aspects of digestive system function, including digestion, metabolism, supporting the innate immunity of epithelial cells, combating pathogens, and facilitating communication between the brain and gut through their adhesion to the human intestines [85,86]. Additionally, probiotics contribute to immune processes by enhancing antibody responses and suppressing the proliferation of mononuclear cells [86]. When combined with fermented non-digestible food products, known as prebiotics, they exhibit various beneficial properties, including anti-pathogenic, anti-inflammatory, antidiabetic, and anti-obesity effects [87,88]. Eleven studies explored the use of prebiotics and probiotics for preventing HPV-induced cervical malignancy [84]. Among them, six studies utilized commercially available topical vaginal prebiotic-containing preparations, while five studies employed preparations containing probiotics, including strains like *Lactobacillus casei*, *L. crispatus*, and *L. rhamnosus*, both alone and in combination with *L. reuteri* [89,90,91,92,93,94]. The probiotic studies included oral formulations containing specific strains and one study on a topical vaginal probiotic preparation [95,96]. The results from the studies on prebiotic preparations were promising, showing increased rates of HPV clearance, cytological and colposcopic clearance of abnormalities, and improved histological outcomes following treatment [89,90,91]. Beyond prebiotic and probiotic preparations, various non-prescription oral and vaginal agents have been explored for potential activity in HPV clearance or the regression of low-grade cervical lesions (Figure 2B). These agents include active hexose-correlated compound, beta-carotene, 3,3′-diindolylmethane, epigallocatechin gallate, indole-3-carbinol, Praneem polyherbal tablets, silicon dioxide with sodium selenite and citric acid, and zinc [97,98,99,100,101,102].

## 5. Link between Cervical Metabolites and HPV Infection

Interestingly, it is not only the CM that may be a predictor of progressive HPV infection. Another large-scale approach used to determine the impact of HPV infections on the development of CIN or cervical cancer is related to the analysis of metabolite composition.

A rapid metabolite screening method using direct-injection mass spectrometry effectively differentiated between cervical cell samples with different early-stage precancerous changes and between samples where hrHPV either cleared or persisted [103]. Importantly, such a discrimination was not influenced by a specific strain of hrHPV but it was due to the presence of the virus itself. Furthermore, the metabolite profiling method was successful in distinguishing levels of low-grade cell abnormalities, a task that is challenging with traditional microscopic screening. This capability has the potential to reduce the misclassification of cases and to minimize costly clinic recalls for women, providing a more accurate classification. Additionally, metabolite profiles could unveil new targets for pharmaceutical interventions aimed at influencing the persistence of HPV infections [103].

Pappa et al. analyzed the metabolic profiles of four distinct cervical cell lines. Those included normal cervical cells and three types of cervical cancer cell lines [104]. Among the cancer lines, one was not infected with HPV (C33A), while the other two were HPV-positive (SiHa with HPV16 and HeLa with HPV18). Sophisticated technologies such as ultra-performance liquid chromatography and high-resolution mass spectrometry were used in this investigation. The results revealed significant differences in metabolites among those cell lines, with from 248 to 326 metabolites showing statistically significant variations. Using random forest analysis, unique molecules were identified for each cell line, highlighting distinct metabolic features. Specifically, both HPV-positive cell lines displayed characteristics consistent with the Warburg effect, a metabolic phenomenon commonly associated with cancer. This suggests that the presence of the HPV E6 protein in those cells influenced their metabolism. SiHa and HeLa cells showed signs of increased activity in the purine salvage pathway, while C33A cells exhibited a novel mechanism involving cytidine synthesis. Overall, these findings shed light on the dynamic and HPV-specific rewiring of metabolic pathways in cervical cancer. Such an approach has the potential to offer new insights into the mechanisms underlying cervical carcinogenesis [104].

Porcari et al. utilized liquid chromatography-mass spectrometry (LC-MS) to identify specific molecular patterns in cervical cytology samples [105]. The LC-MS analysis revealed distinct molecular signatures for high-grade SIL (HSIL), including two ceramides and a sphingosine metabolite. Importantly, those molecules were consistently present, regardless of whether the women had an HPV infection, and they might be linked to the precancerous characteristics of the lesions. Statistical models based on these findings could accurately classify and distinguish women with HSIL from those with no cervical lesions. The results suggested that LC-MS had the potential to become an emerging technology for clinical use in cervical cancer screening [105].

## 6. Next-Generation-Sequencing-Based Studies and the Cervical and Cervico-Vaginal Microbiota

Next-generation sequencing (NGS) has revolutionized the study of the CM and CVM. NGS is a high-throughput DNA sequencing technology that allows for the analysis of large amounts of genetic information from diverse microorganisms present in the cervix [50,106,107,108,109,110,111,112,113,114,115,116,117,118,119]. The technology has significantly advanced our understanding of the complexity and diversity of the cervical microbiota and its implications for health and disease. This high-throughput method allows for the analysis of a vast number of microbial species and their relative abundances in a sample. It provides a comprehensive view of the diversity and composition of microbial communities [120]. NGS data may be used to classify and categorize microbial species based on their genetic sequences. This taxonomic classification helps to understand the prevalence and abundance of specific microbes in the CM and CVM [120]. NGS captures changes in the CM and CVM over time, allowing researchers to study the dynamic shifts in microbial communities during HPV infection or treatment interventions [110,115,119]. Furthermore, NGS-based studies offer personalized insights into an individual’s CM and CVM, potentially guiding personalized approaches to cervical health management and preventive strategies [121]. The knowledge gained from NGS studies of the CM and CVM holds promise for developing novel diagnostic tools, targeted therapies, and preventive interventions for cervix-related conditions, including cervical cancer. The 16s rRNA sequencing is a specific application of NGS that focuses on studying the microbial diversity within a sample by targeting the 16S rRNA gene [122].

The *16S rRNA* gene is present in all bacteria and archaea and contains both highly conserved regions shared among all species and variable regions unique to specific bacterial taxa [123]. By sequencing and analyzing these variable regions, researchers may identify and classify the bacteria present in a sample, even if the organisms cannot be cultured in a laboratory [122].

## 7. Evidence from NGS-Based Studies

In this article, we presented a review of the literature on the relationship between HPV infection and changes among the CM and CVM from studies conducted in the period 2012–2023 using NGS technology (Table 1).

HrHPV infection is the most important factor responsible for cervical cancer development. Nevertheless, the relationship between HPV infection and cancer development is complex and not fully elucidated. For instance, the development of premalignant lesions is associated with a persistent HPV infection. However, to date, the reasons for HPV persistence in some women and clearance in others have not been fully understood. Although the persistence of hrHPV infection is crucial for cervical cancer development, the risk for cancer progression is diverse among women with persistent HPV infection. It was hypothesized that a certain profile or disturbances of the CM and CVM might be associated with HPV-related cervical cancer development. Knowledge of the microbiome promoting a persistent HPV infection or, on the contrary, having a protective effect against HPV could prove extremely useful in adopting strategies for primary prevention. This review paper summarizes the studies on the association of the CM and CVM with HPV infection, precancerous cervical lesions, and cervical cancer.

Based on the analyzed literature (Table 1), significant differences were observed in the cervicovaginal microbiome between the HPV-positive and HPV-negative women. Generally, a wider variety of bacterial species was observed in the HPV-positive women, primarily due to the abundance of species other than *Lactobacillus* and a shift towards anaerobic bacteria [31,36,41,111,117,130,133,135,136,137,139,143]. The majority of the studies indicated that HPV-negative women had a normal cervical microbiota characterized by the abundance of *Lactobacillus*. In addition, several authors suggested that the diversity of CM and CVM correlated with the severity of CIN lesions [107,110,113,115,116,125,128,131,143,144,145] and that bacterial diversity was negatively associated with HPV clearance [134]. These results suggest that the cervical microbiota may have a significant influence on cervical cancer development.

Besides CM and CVM diversity, the studies revealed specific microbes related to persistent HPV infections and CIN development. The majority of the studies indicated *Gardnerella*, *Prevotella*, and *Megasphaera* as species associated with an HPV infection and CIN [36,108,111,117,128,130,131,132,134,145,146]. The mechanisms through which certain microbiota interfere with HPV infections demonstrated in in vitro experiments include the disruption of the cervical epithelial barrier by regulating adherence junction proteins, cervical immune responses, and miRNA expression [108]. The abundance of the bacterial species in the cervical microbiota makes it challenging to indicate one or a few species responsible for a persistent HPV infection and CIN development, and it seems that interactions between cervical microbiota and HPV may be more complex. For instance, Huang et al. [127] found that *Oribacterium*, *Lachnobacterium*, and *Thermus* in the cervicovaginal microbiota were more likely to be associated with HPV-16, while *Motilibacter* was related to HPV-52. Moreover, they reported that the composition of *Litorilinea* and *Paludibaculum* with a concomitant paucity of *Lactobacillus iners* was more likely to be associated with HPV-58. The results indicated that certain bacterial species were more likely to coexist with particular HPV types in the cervical epithelium.

Numerous studies demonstrated that the abundance of *Lactobacillus* in the cervicovaginal flora was correlated with HPV clearance [125,139]. However, the roles of various *Lactobacillus* species may be different. Contradictory results were reported on *Lactobacillus iners*—it was reported to be correlated with HPV clearance by some authors [106,131], while others found its association with HPV persistence [41,108] and CIN-2+ lesions [112]. Such discrepancies may be due to the different study populations, the site of sample collection (cervical vs. vaginal swab), and the detection method used. Furthermore, in the study by Arokiyaraj et al., *Lactobacillus johnsonii* was related to HPV persistence, while *Lactobacillus crispatus* was predominant in the subjects with HPV clearance [128].

Several studies investigated the influence of the CM and CVM on the progression of HPV-related cervical lesions. Overall, the paucity of *Lactobacillus* spp. and increased microbiota diversity were associated with CIN occurrence and progression to cervical cancer [110,127,132], with *Gardnerella vaginalis*, *Prevotella*, *Megasphaera*, and *Atopobium vaginae* being particularly indicated [111,116,117,131,140]. According to Zhang et al. [118], the cervical microbiota may affect cervical cancerogenesis directly and indirectly by affecting the natural history of cervical HPV infection. In the above-mentioned study, the authors observed indirect effects of *Pseudomonas stutzeri*, *Bacteroides fragilis*, *Lactobacillus delbrueckii*, *Atopobium vaginae*, and *Streptococcus agalactiae* mediated by an HPV infection on CIN status. However, direct effects (association with CIN-2+ development) were related to a decrease in the abundance of *Pseudomonas stutzeri* and *Atopobium vaginae*. In addition, the authors observed that in the case of *Pseudomonas stutzeri*, the direct and indirect actions were opposite. This suggests that the interplay between the cervical microbiota, HPV infection and cervical cancer development is complex.

The composition of the CM and CVM is dynamic and alters with time. This was reflected in several studies that compared the microbiome of patients before and after the treatment of cervical lesions with a loop electrosurgical excision procedure. A tendency towards an increase in *Lactobacillus* spp. and a less diverse bacterial environment was observed after the surgical treatment of CIN lesions [108,110,115,119]. These findings bring further questions as to whether the disruption of the CM and CVM is the cause or the effect of HPV infections. In light of the available evidence on the significant differences in the CM and CVM between women with the clearance of HPV infection and those with a persistent infection [125,131,147,148,149], it seems that certain microbiota impact the course of a previously acquired HPV infection rather than impact the probability of HPV acquisition. In other words, the clinical implications of an HPV infection may be determined by the cervical microbiota. Furthermore, a study by Ravilla et al. suggested that the response to the HPV vaccine might be related to the content of the CM [129]. Therefore, the knowledge of a specific microbial environment promoting the progression of CIN lesions could help to identify women at the highest risk of cervical cancer. Furthermore, modifications to the CM could be used as a therapeutic measure to boost HPV clearance.

As regards the limitations of this review article, the retrospective nature and a limited number of samples in most of the studies have to be mentioned. Furthermore, ethnicity might be another confounding factor. Vikramdeo et al. observed differences in the microbiota among women with cervical preneoplasia originating from various racial groups, and the authors suggested that this might explain why certain racial groups differed in terms of HPV incidence and the risk of progression [138].

## 8. Conclusions

Several studies indicated that abnormal CV and CVM might participate in HPV-related cervical cancer development. This implies several clinical issues. For instance, the investigation of the CM and CVM may be used as a part of screening for cervical cancer. However, one of the limitations of using microbiomes for screening purposes is that they are dynamic and subject to constant change. Even if a population at a low risk of cervical cancer could be identified, their cervicovaginal environment could alter towards a high-risk pattern over time. In addition, the treatment of abnormal cervical microbiota may be useful for the management of HPV infection and CIN. However, this requires further prospective trials to evaluate the impact of the above-mentioned intervention. Finally, it is important to evaluate whether the alterations in the microbiome could be a consequence of HPV infection and/or precancerous cervical lesions rather than a factor influencing the course of HPV infection (persistence/clearance). Further large-scale investigations are needed to verify the role of the microbiome in HPV infection and HPV-related cervical lesions.

## Figures and Tables

**Table 1:** Data extracted from studies using next-generation sequencing technology to investigate the relationships between cervical and cervico-vaginal microbiota and HPV infections.

Author, Year, Country	Study Aim	Groups		Material and Detection Method	Results	Changes in Microbiota Abundance	Conclusions
		Cases *N*, (Age)	Controls *N*, (Age)				
Smith et al., 2012, USA [124]	Evaluation of methodological variables of cervical microbiome analysis performance and stability of cervical microbiome collected annually over a period of 5–7 years.	10 HPV+ women in the Natural History Study of HPV in Guanacaste, Costa Rica		Cervical swabs V6 and V6–V9 regions of the 16S rRNA gene; Sanger, Roche 454, and Illumina HiSeq 2000	Ns. differences in age, disease stage, HPV subtype, microbiota community types, and diversity between the HPV-cleared and HPV-uncleared groups. Women with depleted enterococcus ASV_62 and enriched *L. iners* at baseline less likely to achieve HPV clearance at month 12. A negative association between high *L. Iners* abundance and HPV clearance in non-operative treatment patients but not in those who received operative treatment.	Increase: HPV+: *Lactobacillus*, *Gardnerella*	The Roche 454 and Illumina sequencing yielded different community type assignments for certain samples. The primary transition between community types was mainly attributed to a shift between *L. iners* and *G. vaginalis*, which were overwhelmingly dominant.
Oh et al., 2015, Korea [111]	To investigate the connection between CIN and the CM identified through pyrosequencing.	70 with CIN (18–65)	50 controls (18–65)	Cervical swabs Pyrosequencing V1–V3 regions of 16S rRNA Roche/454 GS Junior system	TheIU number was higher in HPV− than HPV+ women.	Increase: *Bacteroidetes*, *Actinobacteria*, *Tenericutes*, *Proteobacteria* higher in HPV+ women; *A. vaginae*, *P. bivia*, *L. fornicalis*, *P. Poae*, and *G. vaginalis* Decrease: *L. iners* and *L. crispatus*	The presence of bacterial dysbiosis, characterized by an abundance of *A. vaginae*, *G. vaginalis*, and *L. iners*, along with a scarcity of *L. crispatus*, in combination with oncogenic HPV, may be a risk factor for cervical lesion.
Piyathilake et al., 2016, USA [112]	To investigate the relationship between the CVM and CIN-2+ in women with well-defined HPV infection and confirmed CIN lesions, considering other risk factors as well.	340 CIN-2 HPV+ (19–50)	90 CIN-1 HPV+ (19–50)	Cervical mucus samples V4 region of the 16S rRNA gene sequenced using Illumina MiSeq	Of the HPV groups, six phyla, *Proteobacteria*, *Firmicutes*, *Actinobacteria*, *Bacteroidetes*, *Fusobacteria*, and *Tenericutes*, were dominant. *Proteobacteria* and *Firmicutes* were the predominant phyla in most women.	Increase: CIN-2 HPV+: *L. iners* and unclassified *Lactobacillus* spp., Lactobacillaceae, *Lactobacillus*, *L. reuteri*, and several sub-genus level *Lactobacillus* OTUs; Bacteroidaceae, Porphyromonadacae, and Coxiellaceae; genera including *Bacteroides*, *Parabacteroides*, *Rickettsiella*, and RFN20	An association between microbiome diversity and CIN severity or oxidative DNA damage was not observed. However, suggestive evidence indicated that CIN-2+ in women infected with hrHPVs might be linked to a cervical microbiome predominantly composed of *Lactobacillus* and *L. iners*.
Audirac-Chalifour et al., 2016, Mexico [106]	To examine the association between CM diversity and composition based on the histopathological diagnosis of each stage of the natural history of CC and the cervical expression levels of IL-4, IL-6, IL-10, TGF-β1, TNF-α, and IFN-γ mRNA.	124 HPV+ (22–61)	81 HPV− (22–61)	Cervical swabs V3–V4 variable regions from 16S rRNA Roche 454, Genome Sequencer Titanium	*L. iners* was the most prevalent species in the cervix among HPV-infected women without lesions.	Increase: HPV+: *P. oleovorans*, *L. iners*, *Sneathia* spp., *S. satelles*, *M. elsdenii*, HPV−: *L. crispatus*, *G. vaginalis* Decrease: HPV+: *G. vaginalis* HPV−: *L. iners*	It is suggested that the CM may be involved in CC pathology. During the development of SIL and CC, some members of the CM could potentially act as modifiers of the cervical microenvironment’s cytokine profile. Accumulating evidence indicates a major role of the microbiota in the immune system modulation of the female genital tract.
Di Paola et al., 2017, Italy [125]	To describe the CSTs linked to HPV-persistence.	27 Clearance—hrHPV infection cleared after one year with no DNA evidence. 28 Persistence—hrHPV infection persisted. (26–64)	17 age-matched HPV− women. (26–64)	Cervicovaginal samples V3–V5 hypervariable regions of 16S rRNA gene Roche 454, GS FLX+ system	*Lactobacillus* was the most abundant genus in CVM. Biodiversity higher in HPV+ group, especially in persistence group vs. control group.	Increase: *L. crispatus*—dominant *Lactobacillus* species in control and clearance groups. Persistence group—higher *Atopobium* levels. Clearance group—mix of aerobic and anaerobic bacteria (*Pseudomonas*, *Brevibacterium*, *Peptostreptococcus*, *Enterococcus*, *Streptococcus*, *Propionibacterium*, *Bifidobacterium*, *Shigella*). Decrease: The persistence group—lower presence of the *Faecalibacterium* compared to the clearance group	Persistence group—low alpha diversity, limited bacterial genera linked to viral persistence. *A. vaginae* abundant, may disrupt epithelial barriers, increasing HPV infection risk. Early CVM characterization identifies high-risk women and informs therapeutic strategies.
Curty et al., 2017, Brazil [126]	To report the initial data on the CM of HIV+ women in the postpartum period. Specific microbiota species as indicators detecting alterations in the cervical microenvironment linked to cervical lesions.	80 women in the Program for HIV-infected Pregnant Women at the Federal University of Rio Janeiro (UFRJ). 25 subjects had samples available at time points of 6 and 12 months. (17–44)	80 women (single timepoint); 26 women samples at the 6-month postpartum time point. In total, 105 individual samples. (17–44)	Cervical cytobrushes V3–V6 regions of 16S rRNA gene Illumina HiSeq 2500 system	The CM of HIV+ women during the postpartum period remained stable, exhibiting a diverse range of bacteria without a dominant presence of *L.crispatus*. Three bacterial genera (*Moryella*, *Schlegella*, and *Gardnerella*) were associated with cervical lesions. Poor knowledge of the functional roles of these bacteria in CM homeostasis and their influence on the development of CC.	Increase: One in high abundance (*Gardnerella*) Decrease: Bacterial genera in low abundance (*Bifidobacterium*, *Moryella*, *Schlegella, and Aerococcus*)	The interaction between the CM, local environment, and immune system is intricate and crucial for maintaining cervical homeostasis. Distinct species within the microbiota function as indicators, detecting changes in the cervical microenvironment, and potentially contributing to its modulation or being influenced by it, leading to either a healthy or diseased state.
Huang et al., 2018, China [127]	To explore the relationship between community composition and single hrHPV-type infection and the relationship between the differentially present microbial species and their effect on hrHPV-type acquisition.	Infected with HPV16, HPV52, and HPV58; both LSIL and HSIL (18–70)	41 healthy women HPV− (18–70)	Cervical cytobrushes V4–V5 regions of 16S rRNA gene, Illumina MiSeq platform	A specific microbial pattern in each hrHPV type was identified, and the crucial microbial species associated with them were characterized.	Increase: HPV16: *Oribacterium*, *Lachnobacterium*, *Thermus* HPV52: *Motilibacter* HPV58: *Litorilinea*, *Paludibaculum* HPV+: *Firmicutes*, *Actinobacteria*, *Fusobacteria*; *P. aquiterrae*, *E. brevis*, *M. indicum*, *A. guillouiae*, *A. citratiphilum*, *L. kribbensis* HPV−: *Proteobacteria*, *B. stagnalis* Decrease: HPV58: *L. iners* HPV+: *Proteobacteria*, *B. stagnalis*, *B. territorii*, *P. mucidolens* HPV−: *Firmicutes*, *Actinobacteria*, *Fusobacteria*	The acquisition of hrHPV does not seem to be influenced by a common CVM group, but rather by specific pathogenic agents unique to each SIL, irrespective of their abundance.
Zhang et al., 2018, Chiny [119]	To investigate the changes in the cervical microbiome after LEEP treatment.	26 HPV+ patients who underwent LEEP for CIN-2 or CIN-3 (25–68)		Cervical swabs V3–V4 hyper-variable regions of the 16S rRNA gene sequenced using Illumina MiSeq	CiRNAseq and 16S rRNA-seq showed similar efficiency in identifying and quantifying microbes. They were in agreement for 81% of the analyzed genera (31 out of 38), demonstrating the high specificity and sensitivity of CiRNAseq at the genus level.	Increase: 3 months after LEEP: *L. iners*; Erysipelotrichaceae and Coriobacteriaceae;Before LEEP: Bifidobacteriaceae, Lachnospiraceae, Leptotrichiaceae, Peptostreptococcaceae, *S. amnii*, *Collinsella*, *Veillonella*, *Clostridia*, *Prevotella*, and unclassified genus belonging to Lachnospiraceae.	In patients with CIN-2/3, LEEP treatment leads to changes in the cervical microbiome. However, LEEP alone is insufficient to fully restore a healthy cervical bacterial community.
Arokiyaraj et al., 2018, Korea [128]	To identify cervical microbes associated with HPV negativity, HPV clearance, and HPV persistence. To assess the longitudinal connections between these microbes and HPV infection dynamics among Korean women.	HPV clearance (42 samples, 15 subjects) HPV persistence (44 samples, 16 subjects) (18–65)	HPV negativity (21 samples, 10 subjects) (18–65)	Cervical cytobrushes V3–V5 hypervariable regions of 16S rRNA gene, Roche 454 GS-FLX plus	Higher diversity was observed in HPV-persistence women compared to HPV− women.	Increase: HPV clearance: the strongest associations with *E. eligens*, *G. vaginalis*, and *U. urealyticum*. HPV persistence: was strongly associated with *L. johnsonii*. HPV+: the highest abundance of *A. vaginae*. Decrease: *L. crispatus* was mainly dominated by the HPV− group	It is suggested that the presence and prevalence of a specific cervical microbiome are factors involved in HPV dynamics. The strongest associations with HPV persistence were observed in women with high proportions of *L. johnsonii*, *Haemophilus* (genus), and Mycoplasmataceae (family).
Zhang et al., 2018, China [118]	To examine the relationships between microbiotas and the severity of CIN, both directly and indirectly.	126 women with CIN-1− (normal cytology and CIN-1); 40 with CIN-2+ (CIN-2 and 3).		Biopsy specimens V3–V6 regions of 16S rRNA gene, Illumina HiSeq 2500 platform	Highly sensitive PCR primer set (SPF1/GP6+) used to detect HPV DNA by amplifying a 184-bp fragment of the L1 open reading frame may still underestimate the proportion of certain HPV infections.	Increase: HPV+: *S. agalactiae*, *B. fragilis*, *P. stutzeri*, and *P. anaerobius*. CIN-2+: *L. crispatus*, *S. agalactiae*, *B. fragilis*, and *C. ureolyticus.* Decrease: HPV−: *L. delbrueckii* CIN-2-: *P. damselae*, *L. jensenii*, and *A. vaginae*	Given the small sample size and the sampling site limited to the location of CIN rather than the entire cervix, the prevalence of HPV infection in our participants may have been underestimated. Considering the limited number of HPV+ samples, the intention was to include a larger number of individuals in future studies to validate our findings.
Ravilla et al., 2019, USA [129]	To assess the potential impact of the cervical microbiome on vaccine response and explore the determinants of the cervical microbiome composition in women diagnosed with high-grade squamous intraepithelial lesions.	31 patients who received vaccination, with biopsy-proven CIN-2/3 (22–49)		Cervical brushes Fragmented and labeled with biotin amplicons of 16S rRNA, hybridized to the PhyloChip Array (version G4)	Ns. difference in HPV contig richness was found by genital inflammation status or microbiome profile.	Between HPV16+ and HPV16- samples, notable variations in beta diversity were found. A total of 15 eOTUs displayed significant differences in their abundances.	Certain bacterial taxa, including *Caldithrix*, *Nitrospirae*, and *Prevotella*, might influence the response to the HPV therapeutic vaccine. However, vaccination did not seem to impact the composition of the cervical microbiome. Race and HPV16 infection seemed to have an influence on the beta diversity of the cervical microbiome.
Onywera et al., 2019, South Africa [130]	To investigate the composition and diversity of CM in reproductive-age black South African women and explore their connections with HPV infections.	37 women (18–65)	50 women (18–65)	Cervical swabs V3–V4 regions of 16S rRNA gene, Illumina MiSeq platform	A total of 28 bacterial taxa were found to exhibit differential abundance between the CM of HPV− and HPV+ women. Neither *Lactobacillus* nor species within this genus were found to be differentially abundant between women with and without HPV or hrHPV infections.	Increase: In comparison to those with lrHPV or no HPV-infection, women with hrHPV displayed significantly higher relative abundances of Aerococcaceae, Pseudomonadaceae, and Bifidobacteriaceae. Furthermore, *Gardnerella*, *Sneathia*, and *Atopobium* were also found to have higher relative abundances in hrHPV-infected women compared to those with lrHPV or HPV−. Decrease: *Campylobacter*, *Haemophilus*, and *Pseudomonas*.	To date, the association between prevalent HPV and CM in a black South African cohort has been examined for the first time. Further investigations into the role of the cervical and vaginal microbiome in HPV/hrHPV infections are warranted.
Ritu et al., 2019, China [113]	To investigate the connections between CM and various HPV infection statuses in women with normal cytology; analysis of the variations in CM linked to the acquisition, persistence, and clearance of different HPV genotypes through a one-year follow-up period.	90 HPV+ (27–65)	43 HPV− (27–65)	Cervical swabs 16S rDNA sequencing with Illumina Hiseq 2500 platform.	Several taxa that could distinguish baseline HPV positivity and predict the acquisition, persistence, or clearance of HPV within a one-year period was discovered. No significant difference in evenness diversity was observed among different HPV infection statuses.	HPV+ women compared to HPV− exhibited higher richness influenced by the abundance of genera other than *Lactobacillus*, including *Acinetobacter*, *Burkholderia*, *Campylobacter*, *Pseudomonas*, *Corynebacterium*, *Halorubrum*, and *Halorientalis*. This richness was found to have the strongest correlation with evenness diversity.	Specific compositions of the CM associated with distinct HPV infection statuses could serve as a biomarker to identify women at risk of persistent HPV infection. Further investigations into the mechanisms underlying these associations may provide valuable insights for developing new therapeutic strategies that modify the microbiota of the reproductive tract to enhance HPV infection clearance.
Usyk et al., 2020, Costa Rica [131]	To investigate the impact of the CVM on the natural history of incident hrHPV infections, focusing on three key aspects: 1. Advancement to cervical precancerous stages; 2. Duration of viral presence in the body; 3. Elimination of the virus from the body (viral clearance).	273 women recruited at first clinical visit (V1)—HPV testing. 266 in follow up examination, at a subsequent visit (V2), meeting criteria for persistence (having the same HPV type at least 305 days after V1), progression (closest visit before diagnosis of CIN2+), or clearance (following visit negative for that type).		Cervical brushes V4 variable region of the 16S rRNA gene, Illumina MiSeq platform		Increase: *L. iners* linked to the clearance of newly acquired hrHPV infections (V1); *Gardnerella* dominant biomarker associated with hrHPV progression.	*Gardnerella* affects the CVM balance, influencing hrHPV progression to precancer. Positive association between *Gardnerella* at V1 and CIN2+ progression was mediated by the increased CVM diversity observed at V2.
Andralojc et al., 2021, The Netherlands [41]	To evaluate the potential of the CVM-specific CiRNAseq assay, validate the technique’s resolution, specificity, and performance in vitro using mock samples, and profile the CVM in a cohort of cervical smears from women with or without hrHPV-associated cervical abnormalities.	46 HPV− women: RNA isolation + CiRNAseq 46 HPV+ women with CIN-2+: RNA isolation + CiRNAseq	10 HPV+ women—DNA isolation + CiRNAseq	Cervical smears CiRNAseq, Illumina NextSeq platform	The top 10 virus genera included: the most dominant Alphapapillomavirus (includes HPV), Betatomopoxvirus, Betabaculovirus, Simplexvirus, Cafeteriavirus, Coccolithovirus, Mimivirus, Betaretrovirus, Ichnovirus, and Alphabaculovirus. HPV16, 32, and 53 were the most prevalent. The HPV-dominated group: 47.62% CIN-1 and 42.86% CIN-2/3 samples; the non-HPV-dominated group: 52.38% CIN-1 and 57.14% CIN-2/3 samples.	Increase: HPV−: *L. acidophilus*, *L. crispatus*, *L. jensenii*, *L. psittaci*, *L. ultunensis*, *L. vaginalis* HPV+: *A. vaginae*, *D. micraerophilus*, *G. vaginalis*, *S. amnii*, *S. sanguinegens*, *L. iners* and *Prevotella* species: *P. amnii*, *P. buccalis*, *P. timonensis* Decrease: After 3 months of LEEP treatment: cervical microbial diversity, *L. amnionii*, and *Clostridium sensu stricto*	CiRNAseq revealed that the the CVM transitions from a healthy *Lactobacillus*-dominated state (CST I) to an anaerobic-diverse state (CST IV) during persistent hrHPV infection. CiRNAseq proves to be a highly promising technology with its high-resolution and specificity for high-throughput sequencing, making it an intriguing tool for exploring the role of CVM in both health and disease.
Wu et al., 2021, China [116]	To examine the cervical microbiome characteristics in reproductive-age women during the transition from SIL to CC.	13 women with CC, 31 HSIL, 10 LSIL, 12 HPV+ (NH) (18–52)	28 healthy controls (NN) (18–52)	Cervical swabs V4 region of 16S rRNA gene, Illumina NovoSeq6000	CC group had the highest community diversity of CM.	Increase: *Prevotella*, *Megasphaera* Decrease: *Lactobacillus*	As the lesions progressed, there was a noticeable upward trend in species diversity.
Zhai et al., 2021, China [117]	Examining the CVM in women of childbearing age with different degrees of cervical lesions and hrHPV positivity.	29 hrHPV+ 32 LSIL 40 HSIL 38 CC (30–50)	29 HPV− women (30–50)	Cervical swabs V3–V4 regions of 16S rRNA gene, IonS5TMXL platform	In the healthy group, *Prevotella* suppressed the abundance of *Lactobacillus*. In the disease groups, *Prevotella* promoted the abundance of *Gardnerella*.	Increase: *Actinobacteria*, *Gardnerella*, and *Prevotella* Decrease: *Firmicutes*, *Lactobacillus*, *Ignatzschineria*, and *Streptococcus*	The healthy group: a strongest association with the genera *Lactobacillus* and *Ignatzschineria*. The disease groups were most closely related to the genera *Gardnerella* and *Prevotella*. A vaginal environment with low abundances of *Lactobacillus* and *Ignatzschineria* might facilitate the progression of lesions into cancer.
Zhang et al., 2021, China [132]	To investigate the similarities and differences between the cervical and vaginal microbiota in hrHPV-infected women in China.	32 of the other hrHPV group (Group O) (25–45)	20 control group (Group N) 38 HPV 16/18 group (Group H) 10 CC group (Group C) (25–45)	Cervical and vaginal swabs V3–V4 regions of the 16S rRNA gene, Illumina MiSeq platform	In the normal group and the hrHPV+ group, hrHPV16/18 infection was associated with higher microbial diversity in the healthy cervix compared to the vagina. HPV− subjects in the normal group exhibited a lower percentage of *Firmicutes* and a higher percentage of *Proteobacteria* in the normal cervix compared to the vagina.	Increase: Cancerous cervix: γ-*Proteobacteria*. Cancerous vagina and cervix: *Prevotella*. HPV16/18(+) CC and the cancerous vagina/cervix: *Gardnerella* and *Atopobium*. All hrHPV-infected vagina/cervix: *Sneathia* irrespective of cancerous status. Decrease: CC: *Lactobacillus*. *Lactobacillus* in cervix compared to the vagina in both hrHPV+ and hrHPV− subjects. However, this difference was not significant in the cancerous cervix.	The findings showed that the cervix and vagina had distinct compositions of the phylum *Proteobacteria*. Specifically, *Sphingomonas*, belonging to α-*Proteobacteria*, demonstrated potential protective effects against hrHPV infection. On the other hand *Pseudomonas*, in the γ-*Proteobacteria* group, showed a positive association with hrHPV infection and CC.
Kawahara et al., 2021, Japan [108]	To investigate the connections between CVM, HPV infection, and cytokine profiles in premenopausal women with CIN before and after undergoing surgical procedures such as laser cone resection, diathermy, and LEEP.	28 Japanese patients with CIN needed surgery, 5 individuals underwent laser cone resection, 23 patients underwent LEEP with diathermy. (24–48)	13 Japanese patients with CIN observation only (24–48)	Cervical swabs V3–V4 regions of the 16S rRNA gene, Illumina MiSeq platform	*L. crispatus* negatively correlated with anaerobic bacteria like *Dialister*, *A. vaginae*, *Adlercreutzia*, *Parimonas*, and *Clostridium* in both collections. Anaerobic bacteria (*Prevotella*, *Dialister*, *A. vaginae*, *Sneathia*, *Adlercreutzia*, *Peptoniphilus*, *Megashpaera*, *Parvimonas*, and *Clostridium*) positively correlated with each other and were unchanged after surgery. *L. crispatus* strongly associated with *L. jensenii* during the first collection and after surgery.	Increase: After surgery: *Tenericutes*, *Ureaplasma* Decrease: After surgery: *Proteobacteria*, *A. vaginae*, and Methylobacteriaceae	Atopobium and *Gardnerella* were associated with HPV and CIN. Reduced HPV infections and neoplastic lesion removal may decrease microbiota diversity. *L. iners* and *Gardnerella* disrupt the cervical barrier, while *L. crispatus* has a protective effect. Proinflammatory cytokines increased with anaerobic bacteria presence and inversely with *Lactobacillus* dominance. Surgical intervention dramatically changed the CVM and local immunity.
Sasivimolrattana et al., 2022, Thailand [133]	To examine the bacterial, fungal, and viral communities in the cervix of Thai patients with HPV16 and high-risk HPV infections at different precancerous stages.	43 patients HPV+ with CIN at different stages: 22 CIN-1, 7 CIN-2, and 14 CIN-3 (23–50)	5 CIN-1 HPV− (23–50)	Cervical swabs V1–V9 region of bacterial 16S rRNA gene; fungal ITS1 and ITS2 genes, Illumina MiSeq platform	Over the period of 5–7 years, the cervical microbiome’s categorical composition exhibited both relative stability and occasional fluctuations between a small number of defined community types. Ns. differences were observed in fungal abundance among the groups.	Increase: CIN-1, CIN-2/3 HPV+: *L. iners*, CIN-1 HPV− NHD: *Parvimonas* sp., *Olsenella* sp. Decrease: CIN-1 HPV−: *C. albicans* CIN-1, CIN-2/3 HPV+: bacterial diversity, human viral diversity	*Lactobacillus* sp. influenced bacterial diversity, and HPV infection impacted both bacterial and human viral diversity. Certain microorganisms showed correlations with HPV infection and dysplasia severity, suggesting their potential as diagnostic tools.
Shi et al., 2022, China [134]	To investigate the association between the CVM at baseline and the clearance of hrHPV infection within 12 months.	45 HPV-cleared after 12 months (24–68)	28 HPV-uncleared after 12 months (24–68)	Cervical swabs V4–V5 regions of 16S rRNA gene, Illumina MiSeq platform	After 12 months, patients with HSIL had slightly higher clearance rates compared to those with HPV+/LSIL, with the difference approaching statistical significance. No significant differences were observed between patients who successfully cleared HPV and those who did not among both α- and β-diversity.	Increase: HPV16 or non-*Lactobacillus*-dominated community state type: higher microbiome diversity; HPV-cleared: Enterococcus ASV_62 (at baseline); HPV-uncleared: *L. iners* (at baseline)	*L. iners* abundance at diagnosis was negatively related to HPV clearance over 12 months, especially in non-operative treatment patients. This highlights the potential role of the microbiota in persistent hrHPV infections.
Liu et al., 2022, China [135]	To explore how the vaginal microbiota contributes to reducing disease risk and identify factors affecting disease susceptibility in six Chinese nationalities (Zhang, Naxi, Yi, Bai, Lisu, and Han).	43 HPV+ (30–50)	39 HPV− (30–50)	Cervical swabs V4–V5 regions of 16S rRNA gene, Illumina MiSeq platform	A potential association between *Prevotella* and cervical disease was indicated.	Increase: HPV−: *Lactobacillus*, *C. accolens*, *M. cohnii*, *R. bromii*, *L. herbarum*, *P. flavescens* HPV+: *C. flavescens*, *C. jeikeium*, *C. ihumii*, *C. gottingense*, *M. mulieris*, *C. acnes*, *P. niger*, *S. chromogenes*, *B. velezensis*, *C. ureolyticus*, *A. johnsonii*, *A. lwoffii*, *P. excrementihominis*, *R. pickettii*, *S. sanguinegens*	Monitoring the microbial environment in the vagina and cervix can help identify early HPV infections and other health issues. Additionally, adjusting the microbial environment offers a potential approach to promoting vaginal and cervical health.
Kaelin et al., 2022, USA [109]	To investigate the relationship between the cervicovaginal DNA virome and other features of the local microenvironment, including CVM and genital inflammation, and examine these factors, which influence HPV persistence and progression to CC.	18 HPV+ premenopausal, nonpregnant women (23–50)	5 HPV− premenopausal, nonpregnant women (23–50)	Vaginal swabs and cervicovaginal lavage V4–V5 regions of 16S rRNA gene, Illumina MiSeq platform	HPV+ groups and certain HPV infections had more diverse microbiota compared to HPV− groups. The age group over 60 also showed higher microbiota diversity. The study highlighted the significant impact of microbiota, particularly pathogenic microorganisms, on metabolic function.	Increase: HPV+: *Alphapapillomavirus*	Anelloviruses were linked to genital inflammation. An association between trans-kingdom interactions, the type of microbiome profile (*Lactobacillus* dominated vs. non-*Lactobacillus* dominated), and genital inflammation. Cervicovaginal virome might play a role in microbiome changes and inflammation, potentially leading to persistent HPV infections and the development of CC.
Hu et al., 2022, China, Australia [136]	To investigate the association between HPV infection and CM changes, especially in relation to different HPV groups and genotypes, and the impact of the microbiota on cellular and metabolic functions; to explore microbiota changes across different age groups within a population cohort in Sanmenxia, Henan Province.	94 HPV+	182 HPV−	Fluid sample after Pap Smear preparation V3–V4 regions of 16S rRNA gene, Illumina HiSeq platform	Predominant microbiota compositions included specific species: *L. iners*, *E. coli*, *E. faecalis*, and A. vaginae. Significant differences in microbiota diversity observed between the HPV+ group and those infected with unique-268 and multi-268 HPV strains compared to the HPV− group. Furthermore, the study revealed that women older than 60 years exhibited higher microbiota diversity compared to younger women.	Increase: HPV+: higher diversity with *Bifidobacteriales*, *Lactobacillus*, Bifidobacteriaceae, *Gardnerella*, *Coriobacteria*, *A. vaginae*, *Clostridia*, and *Sneathia*. Unique-268 HPV+: *Betaproteobacteriales*, Burkholderiaceae, Weeksellaceae, *Flavobacteriales*, *Gardnerella*, *P. aeruginosa*, and *Mycoplasma* compared to multi-268 HPV+. Multi-268 HPV+: Presence of *Saccharimonadales*, *Saccharimonadia*, *Patescibacteria*, *Bifidobacteriales*, and Bifidobacteriaceae.	Increased microbial diversity and a higher proportion of pathogenic microorganisms are likely associated with abnormalities in metabolic functions. The clinical implications of the above microbiota results under different HPV infection statuses involve the identification of potential biomarkers for diagnosing cases.
Molina et al., 2022, The Netherlands [31]	To characterize CSTs in samples from hrHPV− women and hrHPV+ women with and without cervical lesions, using ciRNAseq for high-resolution CVM profiling.	200 HPV+ samples, divided into 100 LSIL and 100 HSIL (CIN-2+) cases; 44 HPV−	297 women without cervical lesions	Cervical smears CiRNAseq, Illumina Nextseq500 platform	Cervicovaginal microbes were categorized into five distinct CSTs, characterized by their microbial community composition and abundance. CSTs I, III, and IV based on intra-CST differences with respect to abundances of L. acidophilus (CSTs I-A vs. I-B and CSTs III-A vs. III-B), L. iners (CSTs I-A vs. I-B and CSTs III-A vs. III-B), and M. genomosp type 1 (CSTs IV-A vs. IV-B). CST V was associated with uninfected conditions, and CST IV-A was associated with hrHPV-induced cervical disease.	Increase: HPV−: *L. acidophilus* hrHPV+: CST IV in NILM, LSIL and HSIL groups Decrease: hrHPV+: *L. crispatus* (CST I) in NILM, LSIL, and HSIL groups; CST V in HSIL	An agreement on CST designation based on high-resolution CVM profiling is promoted, considering microbial dominance, composition, abundance, and diversity. Microbial dynamics occurring in the CVM are suggested by this classification. The data emphasize the identification of commonly overlooked bacterial species, such as *L. acidophilus* and *M. genomosp* type 1, which are relevant for cervical health and microbial relationships. High-resolution microbiome profiling for appropriate classification is necessary.
Fang et al., 2022, China [107]	To examine the composition and function of the CM and its association with hrHPV infection and find ways to prevent persistent hrHPV infection by restoring a healthy microbial balance in the reproductive tract.	20 hrHPV+ (25–45)	20 hrHPV− (25–45)	Cervical swabs V3–V4 regions of 16S rRNA gene, Illumina Novaseq 6000 platform	The study highlighted significant differences in the cervical microbiome between hrHPV-infected and uninfected women. Notably, three species, *L. crispatus*, *L. jensenii*, and *L. helveticus*, stood out as potential microbial targets for future treatment due to their biomarker significance.	Increase: hrHPV+: *Gardnerella*, *Atopobium*, and *Bifidobacterium* hrHPV−: *L. crispatus*, *L. jensenii*, *L. helveticus* Decrease: hrHPV+: *Lactobacillus*, *L. crispatus* hrHPV−: *Gardnerella*, *Atopobium*	By utilizing both 16S rRNA gene and metagenomic sequencing, a comprehensive understanding of the diversity, composition, and function of CM was achieved.
Li et al., 2022, China [110]	To examine the CVM before and after treatments and explore its association with HPV persistence.	26 HPV16+ and CIN-1, 34 HPV16+ and CIN-2/3, 6 HPV16+ and squamous cell carcinoma. (<29 and >60)	25 healthy controls (<29 and >60)	Cervical swabs V3–V4 regions of 16S rRNA gene, MiSeq Illumina platform	*Firmicutes*, *Bacteroidetes Proteobacteria*, *Actinobacteria*, and *Fusobacteria* were dominant. Following clinical treatment, there was a tendency towards increased abundance of *Lactobacillus* and decreased abundance of non-*Lactobacillus* genera.	Increase: The dominant bacteria in CST2 and CST4, such as *Burkholderia*, *G. vaginalis*, *Pseudomonas*, *E. coli*, *Atopobium*, *S. amnii*, and *Prevotella*, were associated with bacterial vaginosis and could potentially contribute to the development of CIN. Decrease: Non-*Lactobacillus* genera, including *Burkholderia* and *Pseudomonas*	The study revealed that advanced CIN lesions are associated with increased CVM diversity. After treatment, a reduced diversity in the CVM was observed in CINs. This suggests that both antiviral and local excisional treatments effectively clear HPV16 infection and aid in the recovery of the CVM.
Guo et al., 2022, China [137]	To examine the differences in CVM among HPV−, HPV+NoSIL, HPV+LSIL, and HPV+HSIL groups; to interpret the association of CVM with HPV infection and SIL level.	40 HPV+NoSIL 28 HPV+LSIL 51 HPV+HSIL (19–50)	30 HPV− (19–50)	Cervical brushes V3–V5 region of 16S rRNA gene, NovaSeq Illumina platform	The analysis at the phylum level revealed higher diversity of taxonomic phylum in the HPV+HSIL group compared to the other three groups. This included increased levels of *Fusobacteria*, *Proteobacteria*, and *Tenericutes*.	Increase: Women with SIL: non-*Lactobacillus* CVM compared to women in the HPV− and HPV+NoSIL groups. HPV+HSIL group: *Megasphaera*. Decrease: HPV+HSIL group: *Enterococcus*.	There were observed significant differences in the CVM among women with HSIL, supporting the association between the CVM and clinical outcomes of HPV infection. Possibly, the CVM may influence the risk of persistence of pre-existing HPV and SIL progression, rather than the risk of HPV acquisition.
Vikramdeo et al., 2022, USA [138]	To analyze cervical intraepithelial lesions from women with different ethnic backgrounds in the United States, i.e., Hispanic/Latina (HIS), African American (AA), and Caucasian American (CA) and their resident microbial compositions, as these groups show variations in CC incidence and outcomes.	36 CIN tissues from various grades (CIN-1-CIN-3). 12 CA, 12 AA, 12 HIS (21–62)		Biopsy specimens V4 region of 16S rRNA gene, MiSeq Illumina platform	Exclusively in women with a histopathological diagnosis of CIN, a unique niche of 27 microbes was identified. A group of 8 microbiota (*Rubellimicrobium*, *Podobacter*, *Brevibacterium*, *Paracoccus*, *Atopobium*, *Brevundimonous*, *Comamonous*, and *Novospingobium*) was exclusively detected in the CIN lesions obtained from AA and CA women.	Increase: *Micrococcus* in AA and HIS compared to CA. *Prevotella* in HIS compared to CA and AA. *Rubellimicrobium*, *Podobacter*, *Brevibacterium*, *Paracoccus*, *Atopobium*, *Brevundimonous*, *Comamonous*, and *Novospingobium* were exclusively detected only in CIN samples of AA and CA. Decrease: *Lactobacillus* in AA and HIS compared to CA	The study identified distinct microbiota abundance in women from different racial groups with cervical preneoplasia. These differences may play a role in diverse CC risk outcomes and disease progression.
Wang et al., 2022, China [139]	To study the CM composition, diversity, and signaling pathways in patients with CIN and CC.	9 CIN-1, 11 CIN-2, 17 CIN-3, and 9 CC samples (22–62)	14 normal samples (22–62)	Biopsy specimens V4 region of 16S rRNA gene, MiSeq Illumina platform	Β-, γ-, and α-*Proteobacteria*, *Bacillus*, and *Clostridium* were the dominant strains in the normal group, CIN, group and CC group. *Lactobacillus* was the dominant strain in each group, although some samples in the normal group did not exhibit dominant *Lactobacillus*. A predictive model was established to assess the potential for malignant transformation from the perspective of cervical microbial genes.	Increase: The normal group: mainly composed of Gammaproteobacteria. CIN-1 and CIN-2 groups: dominated by Sphingomyces. CIN-3 and CC groups: predominantly composed of *Bacteroides*.	The close relationship between vaginal microecology and CIN was established. This study identified key genes from the cervical microbial community associated with CIN’s occurrence. An early warning model was established, which includes the *ABCG2+PCNA+TDG* genes and offers a target for clinical prediction and intervention to prevent the malignant transformation of CIN through cervical microbiological-related genes.
Liu et al., 2022, China [140]	To explain the connections between various bacterial species and the expression of HPV oncogenes at distinct stages of CC.	40 hrHPV and CIN (50.00±9,95) 41 CC (54.20±7.79)	34 hrHPV without CIN (49.74±11.49)	Cervical brushes Shotgun metagenomics, Illumina HiSeq 2500 platform	Positive correlation between the presence of HPV oncogene expression and specific bacterial species, particularly within the *Sneathia* and *Peptostreptococcus* genera. Significant increase in aerobic and anaerobic bacteria, as well as a notable rise in both prevalence and expression of HPV E6/E7 oncogenes. Clear decline in the abundance of *Lactobacillus* genus and species, along with the severity of cervical lesions.	Increase: HPV+: *Lactobacillus* genus (65.96% in HPV, 27.81% in CIN, and 9.19% in CC), *Gardnerella* genus (7.81%, 24.25%, and 11.24%), *Prevotella* genus (2.50%, 6.92%, 11.69%) in comparing to CIN and CC Decrease: HPV (+): *L. iners* (33.57%, 18.59%, and 7.47%, respectively) and *L. crispatus* (25.73%, 6.99%, and 0.82%, respectively), *G. vaginalis* (7.72%, 23.85%, 11.11%, respectively), *P. bivia* (0.54%, 3.09%, 6.86%) comparing to CIN and CC	A notable decrease in the abundance of *Lactobacillus* genus and species, coupled with an increase in both anaerobic and aerobic bacteria with an elevation of HPV E6/E7 and the expression of oncogenes observed along with the severity of lesions of the cervix. The overexpression of HPV oncogenes showed associations with specific bacterial species at different stages of CC.
Stoian et al., 2023, Romania [35]	To characterize the CVM in cervical lesion progression and HPV infection status.	76 HPV+ including: 17 ASCUS, 13 ASCH, 18 LSIL, 10 HSIL, 9 SCC, 9 NILM	11 HPV−	Cervical swabs V3–V4 regions of 16S rRNA gene, MiSeq Illumina platform	Unique pattern in a specific group regarding Lactobacillus species, different from other populations. Presence of *L. iners* with the absence of *L. crispatus*, along with *Atopobium* spp., *Prevotella* spp., and *Gardnerella* spp., could be indicative of severe cervical lesions. Strong link between microbiota diversity, HPV infection, and the progression of cervical lesions.	Increase: HPV+: NILM: *L. iners*; ASCUS: *Lactobacillus* unclassified; HSIL: *Gardnerella*; SCC: *Prevotella*; ASCH and SCC: *E. coli* cft073; LSIL: *E. faecalis*. HPV−: higher frequency of the Lactobacillales Decrease: HPV+: SCC: *Lactobacillus*	HSIL and SCC exhibited higher microbiota diversity in comparison to those with NILM results. Absence of *L. crispatus* and the presence of *L. iners* in HPV− individuals with normal Pap results. Among HSIL patients, a few cases demonstrated the presence of *Sneathia* spp. with relatively low numbers, while *Gardnerella* spp. and *E. coli* were the predominant components of their microbiota.
Vargas-Robles et al., 2023, Puerto Rico [141]	To assess differences in the CVM in Puerto Rican women, pregnant, nonpregnant, or menopausal, with or without HPV infections.	133 from total of 294 hrHPV+, including 84 nonpregnant 21 pregnant 28 menopause	74 from 294 HPV−	Cervical swabs V4 region of 16S rRNA gene, MiSeq Illumina platform	CVM was dominated by *L. iners*. Pregnant women in second and third trimesters showed a reduction in diversity and abundance of bacteria SCC non-*Lactobacillus*-dominant linked to bacterial vaginosis. Postmenopausal women displayed higher alpha diversity and a greater proportion of facultative and strictly anaerobic bacteria. Greater alpha diversity was associated with cervical lesions, but no significant associations were found between the microbiota and HPV infection, regardless of whether it was high-risk or low-risk HPV types.	Increase: hrHPV+: *Clostridium* spp. hrHPV+ LSIL: *E. coli* Decrease: hrHPV+: *Ureaplasma*	Women in Puerto Rico typically had a CVM dominated by *L. iners* or a diverse microbial profile, irrespective of their life stage. A high prevalence of hrHPV and less stable bacterial profiles might contribute to the increased risk of CC observed in the population.
Teka et al., 2023, Ethiopia [142]	To analyze and describe the CVM in women with premalignant dysplasia or invasive CC in comparison to healthy women.	93 HPV+, including 60 CC patients without any treatment	27 HPV−	Cervical brushes/swabs V4 region of 16S rRNA gene, MiSeq Illumina platform	Patients with CC exhibited higher alpha diversity compared to individuals with dysplasia and healthy women. Significant differences in beta diversity when comparing CC patients with the other groups. The microbiota composition varied between the dysplasia and CC groups.	Increase: HPV+: *Porphyromonas*, *Peptoniphilus* HPV+ CC: *L. iners* Dysplasia and HPV−: *Lactobacillus* CC: *Porphyromonas*, *Prevotella*, *Bacteroides*, *Anaerococcus*	The diversity and composition of the CVM increased from dysplasia to cancer. Women with dysplasia had higher levels of *L. iners* compared to healthy women.

ASC, atypical squamous cells; ASCH, ASC and high-grade lesions cannot be excluded; ASCUS, ASC of undetermined significance; CC, cervical cancer; CIN, cervical intraepithelial neoplasia; CiRNAseq, circular-probe-based RNA sequencing; CM, cervical microbiota; CST, community state type; CVM, cervico-vaginal microbiota; HPV, human papillomavirus; HPV+, HPV positive; HPV-, HPV negative; hrHPV, high-risk HPV; lrHPV, low-risk HPV; SIL, squamous intraepithelial lesion; LSIL, low-grade SIL; HSIL, high-grade SIL; LEEP, loop electrosurgical excision procedure; NHD, non-HPV-dominated; NILM, negative for intraepithelial lesion or malignancy; Ns., not significant; OTU, operational taxonomic unit; SCC, squamous-cell carcinoma.

## Data Availability

Not applicable.
